# The Role of Goals, Outcome Expectations, and Normative Beliefs in the Occurrence of Aggressive Behavior in Children and Adolescents

**DOI:** 10.1007/s10567-025-00529-3

**Published:** 2025-06-04

**Authors:** Walter Matthys, Dennis J. L. G. Schutter

**Affiliations:** 1 Department of Clinical Child and Family Studies, Utrecht University, Heidelberglaan 1, 3584 CS Utrecht, The Netherlands; 2 Department of Experimental Psychology, Helmholtz Institute, Utrecht University, Heidelberglaan 1, 3584 CS Utrecht, The Netherlands

**Keywords:** Aggression, Children, Cognitive behavioral therapy, Goals, Normative beliefs, Outcome expectations, Schemas

## Abstract

Goals, outcome expectations, and normative beliefs constitute schemas which are thought to affect social information processing and behavior. The aim of this review is to enhance the theoretical framework that elucidates the role of schemas in the occurrence of aggressive behavior in children and adolescents. Empirical and meta-analytic studies on goals, outcome expectations, and normative beliefs in children and adolescents with aggressive behavior are first discussed. Next, areas for future research are specified, in particular the mechanisms involved in the relation between social experiences, schemas, social information processing, and aggressive behavior. According to extant research, we suggest that schemas help elucidate the impact of aggressive children’s and adolescents’ social experiences on their social information processing and, ultimately, their behavior. Therefore, we consider how schemas can be integrated in cognitive behavioral therapy with the objective of achieving long-term changes in adaptive social behavior among children and adolescents with aggressive behavior.

## Introduction

The term “aggression” can be defined as any behavior that is aimed at harming or injuring another person (Parke & Slaby, 1983). The concept of aggression can be further delineated into distinct forms, including physical, verbal, and relational aggression, and distinct functions such as proactive/instrumental and reactive/defensive aggression (Dodge et al., 1997; Vitaro et al., 2006). Aggression is associated with antisocial behavior, which is defined as any action that violates basic norms (e.g., lying violates the norm to speak the truth), rights (e.g., stealing violates the right of others’ properties), and rules (e.g., truancy involves breaking the rule to attend school). When antisocial behaviors constitute legal violations, they are referred to as delinquent behaviors.

Dysfunctional social cognition is one of the underlying factors of aggressive behavior in children. According to the social information processing model of Crick and Dodge (1994), children’s social cognitive functioning occurs in five processing steps: (1) encoding of cues (both internal and external), (2) interpretation of cues, (3) clarification of goals, (4) response access or construction, (5) and response decision. A study by Dodge et al. (2002) provided psychometric support for the Crick and Dodge (1994) model. The study demonstrated, in particular, the psychometric soundness and convergent, discriminant, and construct validity of key constructs from social information processing theory, specifically those pertaining to interpretation, goals, response generation, and response evaluation. 

Crick and Dodge (1994) emphasize the necessity of considering the role of children’s social experiences, both negative and positive, in social information processing. They propose that a mental representation of a past event is stored in long-term memory. This memory becomes integrated with other memories to form a general mental structure or schema (e.g., the normative belief that aggression is acceptable and appropriate) that serves to guide the processing of future social cues. In particular, a schema (e.g., only harsh solutions work) can be activated in current social situations by situational cues (e.g., when a child has said something bad first). In this way, a schema affects the processing of social information. A focus on a limited number of schemas is consistent with research in cognitive science indicating that individuals, when confronted with the overwhelming amount of stimulus information that is present in most situations, often rely on schemas to simplify the cognitive tasks involved in processing this information (Crick & Dodge, 1994; Fiske & Taylor, 1991).

**Fig. 1:**
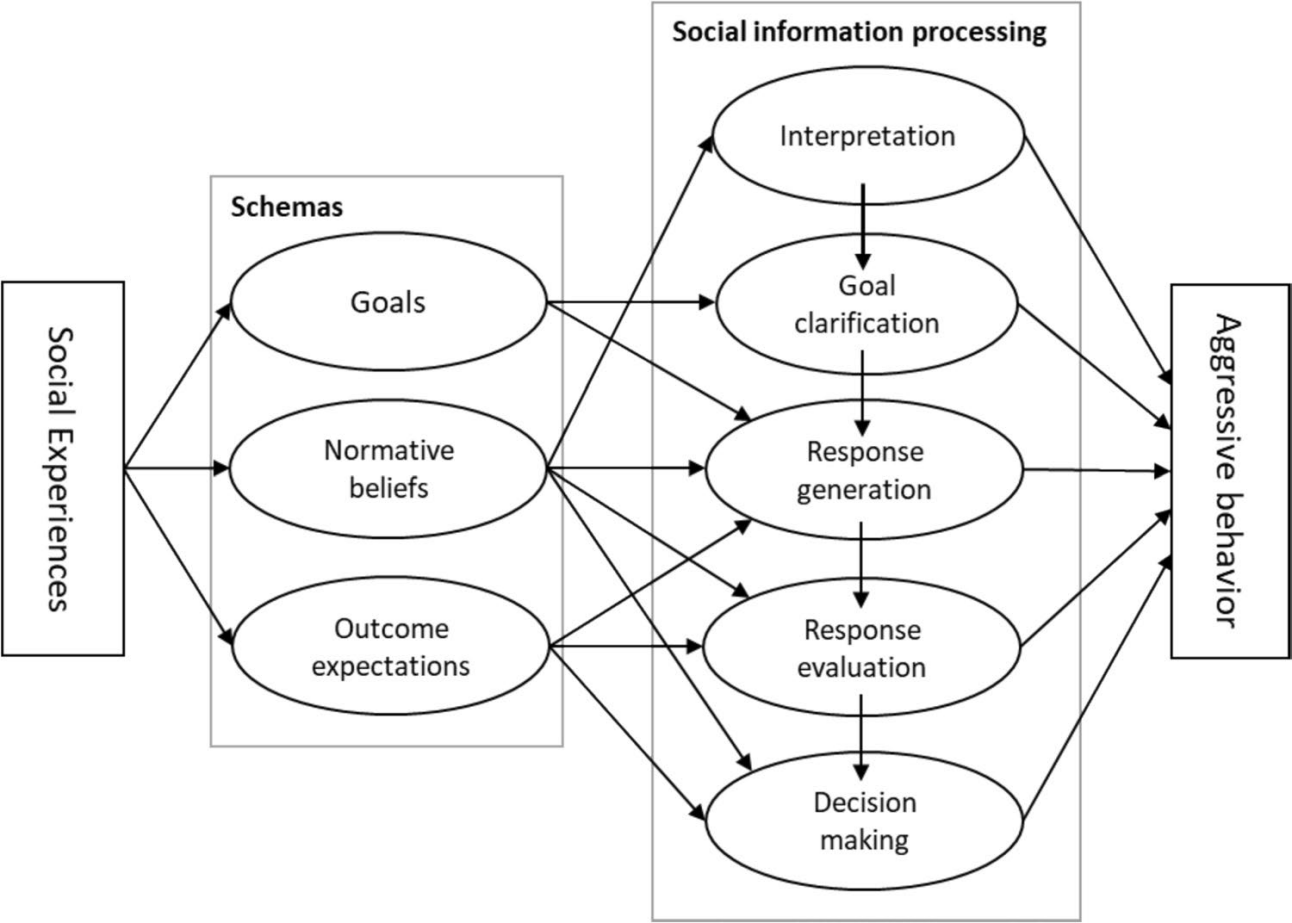
Model of relations between social experiences, schemas, social information processing, and aggressive behavior

The present review will address three schemas: Goals, outcome expectations, and normative beliefs. The aim is to enhance the theoretical framework surrounding the role of schemas in the occurrence of aggression. Social cognitive studies on the three schemas in children and adolescents with aggressive behavior will be first reviewed. Second, we discuss the mechanisms involved in the relation between social experiences, schemas, social information processing, and aggressive behavior. We also identify areas for future research. Third, we consider how schemas can be integrated in cognitive behavioral therapy with the objective of achieving long-term changes in adaptive social behavior among children and adolescents with aggressive behavior. Figure 1 provides a schematic overview of the model which depicts children’s and adolescents’ experiences, schemas, social information processing, and behavior. The model posits that experiences give rise to schemas, which in turn affect social information processing, and subsequently social information processing affects behavior.

## Goals

### Definition and Assessment

Austin and Vancouver (1996) define goals as internal representations of desired states, where states are broadly construed as outcomes, events, or processes. According to the authors, a latent perspective on goals (i.e., goals as latent mental structures) holds that goals define the pursuits of individuals, regardless of awareness or volition. Thus, latent goals are not necessarily beyond awareness, but phenomenal experience is not a prerequisite (Austin & Vancouver, 1996). According to Crick and Dodge (1994) goals, as a social information processing step, are focused arousal states that function as orientations toward producing (or wanting to produce) particular outcomes. As such, goals may include both internal (e.g., feeling happy, avoiding embarrassment) and external (e.g., being first in line at the water fountain during a hot day) states or outcomes (Crick & Dodge, 1994). In addition, these authors propose that children bring goal orientations or tendencies to social situations, here defined as goal-related schemas. Furthermore, children amend those goals and construct new goals in response to immediate social stimuli, here defined as goals as a social information processing step (see Fig. 1).

To study goals as schemas, a questionnaire on goals was developed (Ojanen et al., 2005), see section *Social cognitive studies on goals using a questionnaire*. To study goals as a social information processing step, hypothetical problem situations are used, see section *Social cognitive studies on goals using hypothetical problem situations*. Among the first studies using hypothetical problem situations, the research by Renshaw and Asher (1983) is relevant because it shows the nature of children’s goals. The study examines the goals of third to sixth grade children who are popular compared to those who are unpopular (i.e., in the context of the study of social competence). Popular (or higher status) children had been found to be more friendly and constructive in their peer interactions, whereas unpopular (and/or rejected) children were found to be more aggressive and disruptive. Pilot testing showed that children are unable to first identify their goals for situations; instead, they typically describe strategies that they would use in the situation. Therefore, Renshaw and Asher’s approach was to first ask for a strategy (or a response) and then to elicit goals by asking children about the purpose or goal behind their proposed strategy. For the latter, the interviewer probed children’s underlying goals by asking, “Why would you do (or say) those things?” or “What are you trying to do by doing (or saying) those things?” Rather than using such a “production condition” (i.e., children are unable to first identify their goals), the studies discussed below used a “recognition condition” in which children had to choose between different goal statements.

### Social Cognitive Studies on Goals Using Hypothetical Problem Situations

In one of the earliest studies of social information processing, goal selection was included in the study (Slaby & Guerra, 1988). The authors examined goal selection in violent juvenile offenders, high-aggressive high school students, and low-aggressive high school students. Adolescents incarcerated for having committed one or more violent criminal acts and adolescents rated by their teachers as high aggressive were more likely to choose a hostile goal rather than a non-hostile goal when solving social problems than adolescents who were rated by their teachers as low aggressive (Slaby & Guerra, 1988). Similarly, among adolescent boys, a consistent association has been found between delinquent, substance-using, and behavioral difficulties on the one hand and endorsement of high goal values for dominance and revenge, and low goal values for affiliation on the other. Dominance was found to be the most sensitive correlate of these negative outcomes (Lochman et al., 1993).

In another study, goals were assessed in children who differed in their behavioral responses (i.e., aggression vs. withdrawal vs. problem solving) to ambiguous provocations (Erdley & Asher, 1996). Fourth and fifth grade children who reacted aggressively to hypothetical situations involving ambiguous provocation endorsed more hostile goals than did both the withdrawn and problem-solving responders. Specifically, the aggressive responders were more focused on defending themselves and punishing the protagonist, and were less concerned with maintaining a relationship with the protagonist or dealing with the provocation in a constructive manner.

Furthermore, in distinguishing between proactive and reactive aggression, Crick and Dodge (1996) showed that 9- to 12-year-old proactive-aggressive children are less likely than their non-proactive-aggressive peers (i.e., reactive aggressive and nonaggressive children) to endorse relationship-enhancing goals during social interactions. Rather, they are more likely to prefer goals that are instrumental in nature and relatively self-enhancing (e.g., obtaining a toy from a peer rather than becoming friends with that peer). The authors noted that aggressive acts typically have great potential for damaging relationships with others. Proactive-aggressive children appear to be less oriented toward relational goals than their peers.

Goals have also been studied in delinquent adolescents with callous-unemotional traits or limited prosocial emotions; these include lack of empathy, lack of remorse or guilt, shallow or deficient affect, and unconcerned about performance (American Psychiatric Association, 2013). Both callous-unemotional traits and prior violence were found to be significantly correlated with a greater endorsement of goals related to dominance, revenge, forced respect, and overall conflict escalation, as well as lower endorsement of goals related to conflict avoidance and relationship building (Pardini, 2011).

Among the sources of goal orientation, Crick and Dodge (1994) mention the role of emotions. For example, feeling angry might serve as an impetus for a retaliatory goal. In addition, the intensity with which children experience emotions and their ability to regulate emotions are relevant to mention here. Children who are overwhelmed by their own emotions may choose hostile goals to mitigate these emotions (Lemerise & Arsenio, 2000).

In this regard, a study is relevant in which first to third grade children who varied in social adjustment were randomly assigned to receive either a happy, angry, or neutral mood induction prior to participating in a social cognitive interview assessing goals in the context of ambiguous provocations (Harper et al., 2010). Children who received angry mood induction focused more on instrumental goals than on social-relational goals. In addition, low accepted-aggressive children were more vulnerable to the effects of angry mood induction, choosing more instrumental goals in this condition than in the happy and neutral conditions. These children also chose more instrumental goals than did high accepted-nonaggressive children in the angry mood induction condition. In addition, compared to children with social-relational goal orientations, children with instrumental goal orientations showed less positive outcome expectations for non-hostile responses to provocation (Harper et al., 2010). In a longitudinal study of 4th through 7th graders, aggressive children who had difficulties in regulating their angry and anxious emotions and inhibiting their behavior, endorsed revenge goals at initially higher levels and continued to endorse revenge goals at higher levels for at least two more years (McDonald & Lochman, 2012). In sum, both studies demonstrate the role of emotion regulation in goal orientation (Harper et al., 2010; McDonald & Lochman, 2012).

If goals as a schema reflect mental representations of past social experiences, an investigation into the impact of parenting on children’s goals is a pertinent endeavor. In a longitudinal study, McDonald et al. (2013) examined the association between fourth grade aggressive children’s report of positive parenting and changes in children’s reported social goals over a one-year period. Children’s reports of positive parenting in the fourth grade were positively associated with affiliation goals and negatively associated with dominance and revenge goals in fifth grade. In addition, parent-reported corporal punishment in fourth grade was positively related to dominance goals in fifth grade for boys, but not for girls. These findings indicate that parenting behaviors affect not only children’s behaviors (i.e., the rationale of parent training), but also children’s social cognitions, particularly children’s goals that are part of their social motivations.

Goals as schemas reflect mental representations of social experiences. In a study by Sypher et al. (2019), the effects of parenting and community violence exposure (i.e., social experiences) on children’s social goals were examined in a sample of 426 monozygotic twin dyads, from both population-based and at-risk families. Comparisons of discordant monozygotic twins can be informative regarding the role of the environment, as differences between monozygotic twins must be environmental in origin (Plomin et al., 2001). The authors found that children’s perception of lower positive parenting and perception of greater harsh parenting were associated with greater endorsement of dominance and revenge goals. Furthermore, self-reported exposure to community violence was associated with greater endorsement of avoidance goals. The study demonstrated that social experiences exert an influence on children’s social goals. In particular, results highlight a direct role of parents and community violence in the development of social goals related to aggression. Furthermore, the results of the study indicate the necessity for cognitive behavioral interventions for children to incorporate social goal formation and for parent training to focus on positive parenting techniques.

A meta-analytic study examined associations between goals using the hypothetical peer situation approach and aggression (Samson et al., 2012). The mean positive correlation between antisocial goals and aggression was 0.15, while an inverse correlation of 0.14 was found between prosocial goals and aggression. Age was not a moderator in the associations between goals and aggression. These findings indicate that an increased likelihood of aggression is associated with a low propensity to endorse prosocial goals and a high propensity to endorse antisocial goals for social interaction. In their discussion of these correlations, Samson et al. (2012) cite comparable correlations between aggression and hostile attributions (*r* = 0.17) (de Castro et al., 2002). Samson and colleagues also refer to the meta-analysis conducted by Yoon et al. (1999) and converted the effect sizes reported by Yoon and colleagues in Cohen’s *d* into correlations. The effect sizes between aggression and social information processing variables were comparable, with *r*-values 0.24 for encoding, 0.19 for interpretation, 0.21 for generation of solutions, and 0.19 for evaluation of solutions. In sum, findings from the meta-analytic study by Samson et al. (2012) extend empirical evidence for meaningful associations among the social information processing constructs and aggression to include goal-aggression associations.

### Social Cognitive Studies on Goals Using a Questionnaire

In contrast with the studies discussed above, which examined goals through the use of vignettes depicting hypothetical problem situations, Ojanen et al. (2005) developed a procedure for the assessment of social goals as schemas or trait-like motivational orientations in preadolescents, namely the Interpersonal Goals Inventory for Children. Interpersonal goals are described as organized around the dimensions of *agency* (or power), reflecting authority and appearing confident versus avoiding arguments and anger by going along with others’ expectations (submission) and *communality*, reflecting the striving for closeness and affiliation with others (communality) versus concealing one’s thoughts and feelings (separation). Sample items for each subscale are as follows: Agentic (“others think you are smart”); Agentic and Communal (“you are able to tell others how you feel”); Communal (“real friendship develops between you and others”); Submissive and Communal (“others accept you”); Submissive (“you are able to please others”); Submissive and Separate (“your peers do not laugh at you”); Separate (“you keep others at a suitable distance”); and Agentic and Separate (“the group does what you say”). The instruction was, “When with your age-mates, how important is it for you that...” Children evaluated each item using ratings ranging from 0 (*no importance for me at all*) to 3 (*very important to me*).

In their 2007 study, Ojanen and colleagues employed this procedure to investigate how typically developing children modify their trait-like goals in response to various social contexts, including conflict, group entry, victimization, and positive situations. The findings revealed that children’s social goals have both trait-like and situation-specific characteristics. Approximately half of the observed variation in agentic and communal goals was attributed to between-subject differences, while the remainder was explained by situation-specific effects. With regard to between-subject differences, for instance, boys endorsed more agentic goals than girls. Additionally, a positive self-perception was found to be associated with a greater number of agentic goals, whereas a positive perception of peers was associated with high levels of communal goals. Concerning situation-specific effects, agentic goals were found to be associated with peer rejection, whereas communal goals were associated with peer acceptance. In the absence of a stressful interaction, children most often aimed for closeness to their peers. They endorsed fewer affiliation aims when they were involved in a conflict and had the least of these aims when they were victimized by peers. The prevalence of agentic goals was highest in the victimization situation, followed by the conflict and positive situations, and lowest likely in the group entry situation. Agentic goals may be adaptive in situations of victimization.

A longitudinal study by Ojanen and Findley-van Nostrand (2014) examined prospective associations between goals, physical and relational aggression, social preference (reflecting likeability), and popularity (reflecting status and power) in adolescents aged 12 to 14 years. First, agentic goals predicted increased relational aggression and communal goals predicted decreased physical aggression. Second, goals moderated the longitudinal links between aggression and popularity: Aggression predicted increases in popularity and vice versa for youth with higher agentic goals, and popularity predicted increases in physical aggression for youth with higher agentic and lower communal goals. The findings indicate that agentic goals and popularity are associated with an increased probability of relational aggression, and when combined with low communal goals, may constitute a risk factor for physical aggression during middle school. The authors propose the targeting of goals as a means of reducing aggression and cite an intervention study that demonstrated success in modifying goals. Indeed, in a study of Second Step, a universal prevention program that includes problem solving, intervention children (fifth and sixth grades) were more likely to prefer prosocial goals than control children; intervention children also behaved less aggressively than control children (Frey et al., 2005).

The procedure developed by Ojanen et al. (2005) was used in a study examining the relationship between narcissism, temperament, and social goals with regard to physical and relational aggression in 12- to 14-year-old adolescents (Ojanen et al., 2012). Dominance goals were measured with the Agentic and Separate goal scale and closeness goals were measured with the Communal goal scale. The results indicated a positive correlation between narcissism and dominance goals, and a negative correlation between narcissism and closeness goals with regard to peer interaction. The findings indicated that narcissism was associated with physical aggression via dominance goals for boys and with relational aggression via dominance goals for both genders.

Ojanen and Findley-van Nostrand (2019) examined affective-interpersonal and impulsive-antisocial dimensions of psychopathy in relation to social goals and forms of aggression in nonclinical youth. Only the affective-interpersonal dimension of psychopathy was uniquely positively associated with agentic (status) goals and negatively associated with communal (closeness) goals. In addition, only the affective-interpersonal dimension was indirectly related to proactive aggression via agentic goals. Aggression in the context of affective-interpersonal psychopathy, but not impulsive-antisocial psychopathy, may be driven by high social status and low closeness goals.

Bullying can be conceptualized as aggressive goal-directed behavior that causes harm to another individual in a context of a power imbalance (Volk et al., 2014). It is therefore pertinent to study the role of goals in the context of bullying behavior among children and adolescents. A meta-analysis investigated the associations between situation-specific or global goals and bullying (Samson et al., 2022). The results indicated that children who engaged in more bullying behavior were more likely to endorse status/power goals and antisocial goals and to disregard prosocial goals. Moderators including goal type (situation-specific vs. global goal), gender, and age were not found to be significantly related to effect sizes.

In another meta-analysis of studies on social goals and bullying, a novel model was tested, namely the Social Goals and Gains Model of Adolescent Bullying and Aggression (Hensums et al., 2023). According to this model, adolescents who have agentic goals (i.e., getting ahead of others) rather than communal goals (i.e., getting along with others) engage in more bullying and aggression (relational and instrumental/proactive). Engaging in bullying and aggression may result in adolescents gaining in popularity (i.e., perceived popularity) but losing in likability (i.e., sociometric popularity). As hypothesized, adolescents’ agentic goals were associated with higher levels of bullying and aggression. In turn, bullying and aggression were associated with higher popularity but lower likability. However, there was no significant association between adolescents’ communal goals and bullying or aggression. The results indicated that agentic goals had a small-to-moderate positive association with bullying and aggression which, in turn, had a small-to-moderate positive association with popularity and a small-to-moderate negative association with likability. In contrast, communal goals were not significantly related to bullying and aggression. In terms of interventions, the authors propose that, rather than reducing agentic goals, interventions should focus on modifying the means by which adolescents pursue their agentic goals. For example, schools can create platforms to students (e.g., sports, music) where they can fulfill their agentic goals by showing their talents and feeling competent, without harming others. In comparison, Ojanen and Findley-van Norstrand (2014) suggested a focus on the goals themselves as a means to reduce aggression.

## Outcome Expectations

### Definition and Assessment

According to the model developed by Crick and Dodge (1994), after the generation of responses, the responses are evaluated on outcome expectations. These expectations pertain to children’s ideas about the probable outcome of a social interaction following the enactment of a behavioral response. Outcome expectations as schemas develop over the years on the basis of children’s experiences. In general, children who exhibit aggressive behavior anticipate that their actions will result in favorable outcomes. This is because they have learned that aggression reduces aversive treatment by other people (see the principle of negative reinforcement and Patterson’s Coercive Theory, 1982).

In order to ascertain outcome expectations of the children, they are requested to describe or evaluate “what would happen” if they responded in a certain way in a given situation. The majority of studies have employed evaluative response formats. For example, in the study by Perry et al. (1986), children were asked to imagine themselves engaging in a specific behavior with a particular classmate and then to indicate their level of confidence that a particular consequence would occur by selecting one of four response alternatives. There were six types of consequences: (i) tangible rewards, (ii) adult approval, (iii) peer approval, (iv) reduction of aversive treatment, (v) victim suffering, and (vi) self-reward. In contrast, in the Crick and Ladd (1990) study, children were instructed to imagine themselves employing the strategy in the given situation and to describe the resulting outcome (i.e., what would happen?). A content analysis of the interview transcripts was employed to develop the coding scheme. The outcome-focus categories were as follows: Instrumental-successful, instrumental-unsuccessful, relational-positive, relational-negative, and sanctional.

### Social Cognitive Studies

In one of the initial studies examining outcome expectations, aggressive children in the fourth to seventh grades showed to be more confident that aggression would produce tangible rewards and reduce aversive treatment by others than were nonaggressive children (Perry et al., 1986). Similarly, adolescents who were incarcerated for committing one or more violent crimes generated fewer consequences for aggression than adolescents who were rated as highly aggressive by their teachers, and adolescents who were rated as highly aggressive by their teachers generated fewer consequences for aggression than adolescents who were rated as low aggressive by their teachers (Slaby & Guerra, 1988).

Boldizar et al. (1989) highlighted the necessity to differentiate between outcome expectancies and outcome values. An outcome expectancy can be defined as an individual’s estimate of the likelihood that a given outcome will occur. In contrast, an outcome value represents the degree to which an individual attaches importance to or cares about a specific outcome. The study found that aggressive children (third through sixth grades) placed a higher value on obtaining some of the rewards associated with aggression, while also placing reduced concern for the potential damaging and punitive consequences of aggression compared to their less aggressive peers (Boldizar et al., 1989).

When distinguishing between proactive aggression and reactive aggression, Crick and Dodge (1996) showed that only 9- to 12-year-old proactive-aggressive children evaluated verbally and physically aggressive acts in relatively positive ways. These findings lend support to the proposition that children exhibiting proactive-aggressive behavior are likely to perceive aggression as an effective and viable means for achieving social goals (Crick & Dodge, 1996). Furthermore, a study of incarcerated adolescent boys aged 13 to 18 years examined whether the relation between aggressive outcome expectations was specific to the proactive subtype of aggression as opposed to the reactive subtype (Smithmyer et al., 2000). Controlling for reactive aggression, a relationship was observed between proactive aggression and the tendency to expect positive outcomes for aggressive actions. The aforementioned conclusion was not supported with regard to reactive aggression. This finding is consistent with the view that proactive aggression is goal-oriented in nature (Smithmyer et al., 2000).

There is also evidence that atypical outcome expectations are related to callous-unemotional traits or limited prosocial emotions. In a study of adjudicated adolescents, higher callous-unemotional traits were associated with increased expectations of positive consequences of aggression (i.e., tangible rewards, dominance) and decreased expectations about the negative consequences of deviant behavior (i.e., punishment) (Pardini et al., 2003). In the study by Pardini (2011) previously discussed in the context of goals, expectancies and values regarding victim suffering following aggression were investigated in delinquent adolescents with callous-unemotional traits. No association was found between callous-unemotional traits and expectations of victim suffering following aggression. However, callous-unemotional traits were significantly associated with lower levels of concern (outcome values) about victim suffering as a result of aggression. The author concludes that adolescents with callous-unemotional traits seem to be aware that their aggressive behavior will cause others to suffer, but they do not care when it does.

In a recent study, Elowsky et al. (2022) examined the outcome expectations and values regarding the consequences of aggression in adolescents with conduct disorder, a childhood disruptive behavior disorder, and the role of callous-unemotional traits and irritability. Callous-unemotional traits were associated with decreased expectations that aggression would result in feelings of remorse and victim suffering, as well as decreased concerns that aggressive acts would result in punishment and victim suffering. Irritability was associated with increased expectations and concerns that aggression would result in dominance and force respect, and with placing value on establishing dominance and forcing respect. The findings of this study indicate that callous-unemotional traits and irritability are associated with different forms of maladaptive outcome expectations and values regarding the consequences of aggression.

Shewark et al. (2024) investigated whether neighborhood characteristics (resulting in social experiences) moderated genetic and environmental influences on 6- to 11-year-old twin pairs’ hostile attributions and positive expectations of aggression. Surprisingly, there was no evidence of moderation for children’s hostile attributions. However, genetic influences on aggressive expectations increased in the presence of neighborhood deprivation and decreased in the presence of protective social processes (e.g., protection of support and help among neighbors) and availability of resources. It can therefore be posited that protective processes occurring within neighborhood, which give rise to social experiences, act as a buffer against the development of aggressive expectations during middle childhood by suppressing the expression of genetic influences on these outcomes. In other words, expectations of aggression can be influenced by social experiences.

In this context, the role of outcome expectations as one of several social cognitive processes involved in a preventive intervention is relevant here. The Fast Track study showed that a multiyear, school-based, indicated preventive intervention that included not only the promotion of children’s social cognitive and social skills but also the improvement of parenting skills and academic mentoring, resulted in a decrease of antisocial behavior. This decrease was mediated by its impact on three social cognitive processes: (1) a reduction in hostile-attribution biases, (2) an increase of the generation of socially competent responses to social problems, and (3) an improvement of the evaluation of the outcomes of aggression as detrimental (i.e., devaluing aggression as effective and acceptable) (Dodge & Godwin, 2013). This study demonstrates that devaluing aggression as effective and acceptable is not only feasible, but also a mechanism of change, and as such, constitutes an important aspect of cognitive-behavioral oriented treatment approaches. It is important to note that the devaluation of aggression as an acceptable behavior can be considered not only as an outcome expectation, but also as a component of normative beliefs.

## Normative Beliefs About Aggression

### Definition and Assessment

Normative beliefs are defined as an individual’s cognitive standards regarding the acceptability or unacceptability of a given behavior (Huesmann & Guerra, 1997). These beliefs are part of the child’s latent mental structures or schemas, which influence the child’s online processing of social cues (Huesmann & Guerra, 1997). Normative beliefs are acquired by children through observation, experience, and tuition they receive from peers, parents, and teachers (Huesmann & Guerra, 1997). Scales have been developed to assess whether children believe a behavior is acceptable. For example, How often do you think (never, sometimes, often, or always) it is okay for a boy, Tom, to hit a girl, Julie, if Julie says something bad to Tom first? (Huesmann & Guerra, 1997).

### Social Cognitive Studies

In their 1988 study, Slaby and Guerra (1988) examined the beliefs supporting aggression in violent juvenile offenders, high-aggressive high school students, and low-aggressive high school students. A comparison of violent juvenile offenders with low-aggressive high school students revealed that the former group was more likely to hold a number of beliefs that support the use of aggression. These beliefs included beliefs that aggression is a legitimate response, increases self-esteem, helps avoid a negative image, and does not cause the victim to suffer (Slaby & Guerra, 1988).

Consistent with this, Huesmann and Guerra (1997) found that individual differences in normative beliefs that aggression is socially acceptable and appropriate in fourth and fifth graders predicted an increase in aggressive behavior by sixth grade. Huesmann and Guerra (1997) hypothesized three ways in which normative beliefs may influence children’s aggressive behavior. First, normative beliefs affect the way in which children perceive or interpret the behavior of others; the more children approve of aggression, the more likely they are to perceive hostility in others, even when no hostility is present. Second, normative beliefs that support aggression cue the retrieval of aggressive scripts for social behavior. In other words, normative beliefs may help generate aggressive solutions to social problems. Finally, if normative beliefs act as a filter to eliminate “inappropriate” behaviors from children’s repertoires, children with normative beliefs supporting aggression are less likely to reject aggressive solutions once they have considered them as solutions to social problems. Thus, normative beliefs are suggested to play a role in the evaluation of possible solutions as part of the response decision step of social information processing.

Huesmann and Guerra’s hypotheses were confirmed in a study by Zelli et al. (1999). Individual differences in third graders’ retaliation approval were found to predict individual differences in fifth graders’ aggressive behavior. Nearly 50% of this effect could be attributed to three social information processing steps: (1) attribution of hostile intentions, (2) generation of aggressive responses, and (3) positive evaluation of aggressive responses (Zelli et al., 1999).

Normative beliefs are constructed over time and depend on social experiences. This was demonstrated in a study examining the effects of witnessing community violence on aggressive cognitions and behaviors (Guerra et al., 2003). Exposure to violence predicted aggressive behavior in both 5- to 8-year-olds and children aged 9- to 12-year-olds. However, the effects on normative beliefs were evident only in the older children. Furthermore, the effect of exposure to violence on aggression in the older children was partially mediated by its effect on normative beliefs.

Moral evaluations of solutions are involved in response decision. A positive evaluation of aggressive behavior, including social acceptability and moral appropriateness of aggression, has been shown to increase the prediction of later antisocial problems from externalizing behavior in early adolescence (Fontaine et al., 2002). The distinction between reactive and proactive aggression may be pertinent in this context. A higher level of proactive aggression in adolescents was associated with a lower degree of moral concern (i.e., denying or minimizing negative consequences for others) regarding one’s aggression (Arsenio et al., 2009).

A cross-sectional study of children with mild intellectual disabilities and aggressive behavior examined the potential role of social information processing as a mechanism linking normative beliefs about aggression and aggressive behavior (van Cappellen et al., 2023). The study demonstrated that children with mild intellectual disabilities who believe that aggressive responses in social situations are normal, are prone to interpret social situations as hostile, to generate and select more aggressive responses, and ultimately to exhibit more aggressive behavior in school.

A longitudinal study of adolescents investigated whether the justification of violence schema predicts social information processing, and social information processing, in turn, predicts aggressive behavior (Calvete & Orue, 2012). The justification of violence schema (e.g., It is OK to hit others when they deserve it) was found to predict access to forms of aggressive behavior when the adolescent imagined himself or herself in an ambiguous social encounter (i.e., response generation), and these thoughts predicted reactive aggressive behavior. The authors concluded that interventions should include the modification of dysfunctional schemas.

Another longitudinal study examined the relationship between justification of violence and child-to-parent aggression. The objective was to assess whether social information processing mediated this association (Orue et al., 2021). The justification of violence schema was found to predict hostile attributions, aggressive response access, and the anticipation of positive consequences of aggressive actions. Moreover, the justification of violence schema was found to predict child-to-parent aggression both directly and through aggressive response access.

In a study conducted by Bellmore et al. (2005), it was found that adolescents who believed in the appropriateness of aggression selected hostile response options that subsequently resulted in physical, verbal, and indirect bullying behaviors. The influence of normative beliefs on adolescents’ aggressive reputations among their teachers and classmates was shown to be indirect, in that adolescents’ response selections mediated the influence of their beliefs on their aggressive reputations. With regard to interventions, the authors commented that short-term effects might be obtained by simply changing response selections, but that longer-term effects almost certainly depend on making changes in the schemas that influence social information processing skills.

A systematic review was conducted of studies of normative beliefs supporting aggression as a potential mechanism underlying the relationship between exposure to community violence and aggressive behavior in adolescents (Pittman, 2023). The majority of studies found evidence that normative beliefs about aggression mediated relations between exposure to community violence and aggressive behavior (Pittman, 2023).

Parents may exert an influence on adolescents’ propensity for aggression through the transmission of messages that encourage and discourage the use of aggressive or nonviolent behaviors. In a recent study, Coleman et al. (2024) investigated the role of adolescents’ beliefs about fighting as potential mediators of the longitudinal relationship between perceived parental messages and changes in physical aggression. Parental support for nonviolence was negatively associated with adolescents’ beliefs supporting reactive aggression and positively associated with adolescents’ beliefs against fighting. Additionally, parental support for retaliation was positively associated with adolescents’ beliefs supporting reactive and proactive aggression, and negatively associated with beliefs against fighting. Parental support for fighting as sometimes necessary was positively associated with adolescents’ beliefs supporting reactive aggression and beliefs that fighting is sometimes unavoidable. Furthermore, beliefs supporting reactive and proactive aggression and beliefs that fighting is sometimes necessary were positively associated with aggression, whereas beliefs against fighting were negatively associated with aggression. In conclusion, parental support for nonviolence and for not fighting may reduce adolescent physical aggression by influencing beliefs about the appropriateness of using aggression for self‐defense and to achieve a goal. In other words, normative beliefs about aggression constitute a cognitive function that can be influenced by parents.

## Increasing the Understanding of the Relations between Social Experiences, Schemas, Social Information Processing, and Aggressive Behavior

### Characteristics of Schemas

Social cognitive research on schemas initially focused on the characteristics of schemas in children with aggressive behavior. This research shows that the goals of aggressive children are characterized as hostile, vengeful, dominant, agentic (reflecting authority and appearing confident), punitive, self-defensive, less concerned with maintaining a relationship, and less concerned with dealing with a provocation in a constructive way (Erdley & Asher, 1996; Lochman et al., 1993; Ojanen & Findley-Nostrand, 2014; Slaby & Guerra, 1988). However, if these goals are characteristic of children with aggressive behavior, this does not mean that they always deliberately consider these goals before acting. In contrast, children have difficulty defining their goals before considering possible responses to problem situations (Renshaw & Asher, 1983). This is consistent with Austin and Vancouver (1996), who argue that goals indicate the pursuits of individuals without their having to be aware of them. In real-life situations, both adults and children are often unaware of their goals. However, these goals may nonetheless be accessible to conscious experience. This also applies to the other two schemas or latent mental structures: Outcome expectations and normative beliefs. These schemas may be accessible to conscious experience in both research settings and real-life situations, and they may play a role in real life, which is why they can be a focus for intervention.

Social cognitive research suggests aggressive children hold the belief that aggression is a legitimate response, does not cause the victim to suffer, and is socially acceptable and appropriate (Huesmann and Guerra (1997); Slaby & Guerra, 1988). The distinction between proactive and reactive aggression is relevant to normative beliefs. A higher level of proactive aggression in adolescents is associated with a lower degree of moral concern regarding one’s actions (Arsenio et al., 2009).

With regard to outcome expectations, social cognitive research suggests that children with conduct problems expect that aggression will produce tangible rewards and will reduce aversive treatment by others (Perry et al., 1986). There is also support for the notion that specifically proactive-aggressive children, as opposed to reactive aggressive children, perceive aggression as an effective and viable means for achieving social goals (Crick & Dodge, 1996; Smithmyer et al., 2000). These findings are consistent with the view that proactive aggression is goal-oriented in nature (Crick & Dodge, 1996; Smithmyer et al., 2000). It is interesting to note that outcome expectations are thus conceptually related to goals.

This is also the case in Blair’s (2022) neurocognitive aggression motivation theory. According to Blair’s (2022) cognitive neuroscience approach to aggression motivation, proactive aggression is carried out with the intention of achieving the desired outcome for the aggressor. Reinforcement-based decision-making studies in children with conduct disorder (i.e., clinical levels of aggression), however, show reduced neural responsiveness to reward which puts them at risk for poor decision-making because response choices are less guided by the expectation that an action will result in reward relative to punishment (Blair et al., 2018). As a result, the aggressive and antisocial behavior of children with conduct disorder may be less instrumental, planned or proactive than it may first seem. This is in line with the study by Smeets et al. (2017) in a large clinical sample which showed that no proactive-only group could be determined. A simple division of proactive and reactive aggressive behavior may be less applicable in these children than in those with lower levels of aggressive behavior, possibly due to associated conditions such as Attention/Deficit-Hyperactivity Disorder or trauma-related disorders.

### The Development of Schemas

The mechanisms underlying schema development have been the subject of several studies. In the study conducted by McDonald et al. (2013), the association between fourth grade aggressive children’s report of positive parenting and changes in children’s reported social goals over a one-year period was examined. Children’s reports of positive parenting in the fourth grade were positively associated with affiliation goals and negatively associated with dominance and revenge goals in the fifth grade. The results demonstrate that children’s experiences result in later characteristic goals. In another study, rather than using a longitudinal design, the effects of parenting on children’s social goals were examined in a sample of monozygotic twin dyads (Sypher et al., 2019). Results indicated that perception of lower positive parenting and greater harsh parenting were associated with a higher likelihood of endorsing dominance and revenge goals. These findings highlight the influences of social experiences on children’s social goals.

Outcome expectations of aggression can be influenced by social experiences as well. This was demonstrated by the study conducted by Shewark et al. (2024), which investigated whether neighborhood characteristics moderated genetic and environmental influences on positive expectations for aggression among twin pairs aged between six and 11 years. The influence of genetics on aggressive expectations was found to increase in the context of neighborhood deprivation and to decrease in the presence of protective social processes and the availability of resources. Finally, the development of normative beliefs also depend on social experiences, as shown in the study by Guerra et al. (2003) examining the effect of witnessing community violence on normative beliefs.

### Relations Between Social Experiences, Schemas, Social Information Processing, and Behavior

Before discussing the relationships among social experiences, schemas, social information processing, and behavior in aggressive children, it is first necessary to clarify the two distinct interpretations that can be ascribed to the term ‘goals.’ As discussed before, according to Crick and Dodge (1994), children bring goal orientations or tendencies to social situations (which we define as goals as schemas) and revise those goals and construct new goals in response to immediate social stimuli (which we define as goals as a social information processing step). Ojanen et al. (2007) investigated how typically developing children modify their trait-like goals (i.e., goals as schemas) in response to various social contexts (e.g., conflict, group entry, victimization, and positive situations). The findings revealed that children’s social goals have both trait-like characteristics (i.e., goals as schemas) and situation-specific characteristics (i.e., goals as a social information processing step). Approximately half of the observed variation in agentic and communal goals was attributed to between-subject differences, while the remainder was explained by situation-specific effects.

In regard to the relationships between social experiences and schemas (Fig. 1), we have previously discussed several longitudinal studies showing effects of social experiences on goals (McDonald et al., 2013; Sypher et al., 2019), outcome expectations (Shewark et al., 2024), and normative beliefs (Guerra et al., 2003).

Remarkably, to the best of our knowledge, no studies have been conducted on the effect of goals as schemas on social information processing steps, while one may expect goals to affect the generation of responses and, ultimately, behavior (Crick & Dodge, 1994). Similarly, no studies have been performed to examine the effects of outcome expectations on the generation of responses, the evaluation of responses, decision-making, and ultimately behavior. Further research in this area is essential to gain a deeper insight into the role of schemas in the occurrence of aggressive behavior.

With regard to normative beliefs, on the other hand, the studies by Huesmann and Guerra (1997) and Zelli et al. (1999) are exemplary. Huesmann and Guerra (1997) found that individual differences in normative beliefs that aggressive forms of behavior are socially acceptable and appropriate in fourth and fifth graders predicted an increase in aggressive behavior by sixth grade. Huesmann and Guerra (1997) hypothesized three ways in which normative beliefs affect children’s aggressive behavior. Their hypotheses were confirmed in a study by Zelli et al. (1999). Individual differences in third graders’ retaliation approval were found to predict individual differences in fifth graders’ aggressive behavior. This effect could be attributed to three social information processing steps: (1) attribution of hostile intentions, (2) generation of aggressive responses, and (3) positive evaluation of aggressive responses (Zelli et al., 1999).

Moreover, the longitudinal study conducted by Guerra et al. (2003) demonstrated that the influence of exposure to violence (i.e., belonging to social experiences) on aggression was partially mediated by its impact on normative beliefs in children aged 9- to 12-year-olds. In addition, Calvete and Orue (2012) investigated whether the justification of violence schema predicts social information processing, and social information processing, in turn, predicts aggressive behavior. The justification of violence schema was found to predict access to forms of aggressive behavior when the adolescent imagined himself or herself in an ambiguous social encounter (i.e., response generation), and these thoughts predicted reactive aggressive behavior.

Regarding decision-making, the final step in social information processing, a longitudinal study has demonstrated that a positive evaluation of aggressive behavior, including social acceptability and moral appropriateness of aggression, increases the prediction of later antisocial problems from externalizing behavior in early adolescence (Fontaine et al., 2002).

### Implications for Interventions

Parent training programs are among the most well-studied psychological interventions for the prevention and treatment of antisocial and aggressive behavior in children and adolescents. According to the most recent meta-analysis of parent training including 241 studies, the overall mean effect size at post-intervention (up to 3 months after the termination of the program) was positive for antisocial behavior (*d* = 0.47) (Beelman et al., 2023). However, the effect size for antisocial behavior decreased in short-term follow-ups (3 to 12 months) to *d* = 0.22 and long-term follow-ups (12 months or more) to *d* = 0.12. One way to achieve sustainable effects of parent training is to combine parent training with cognitive behavioral therapy (Matthys & Schutter, 2022).

Children with conduct problems derive increasing benefit from direct participation in cognitive behavioral therapy with increasing age, wherein social problem solving is targeted (Fairchild et al., 2019). Indeed, in the meta-analysis of cognitive behavioral therapy by McCart et al. (2006), a positive relationship between age and effect size was found, showing that as youth ages and progresses into more advanced levels of cognitive development they benefit more from cognitive behavioral therapy. According to British guidelines, group social and cognitive problem-solving programs for the treatment of conduct problems should be based on a cognitive-behavioral problem-solving model (National Institute for Health and Clinical Practice (NICE), 2013, 2017; Pilling et al., 2013). We consider the psychological functions in the social information processing model developed by Crick and Dodge (1994) similar to social problem-solving skills (Matthys & Schutter, 2022, 2023).

Based on a social problem-solving model of cognitive behavioral therapy (Matthys & Schutter, 2022, 2023) and the partially proven effect of schemas on social information processing steps, we here offer suggestions how therapists can assist children and parents in changing children’s goals, outcome expectations, and normative beliefs. There is evidence that schemas, in particular outcome expectations, can be modified and also are a mechanism of change of antisocial behavior (Dodge & Godwin, 2013).

Cognitive behavioral therapy sessions often are offered in a group format. In view of the child’s use of the social problem-solving skills and associated schemas in their daily lives, it is imperative to engage the parents in the intervention process. Schemas are slow to change psychological phenomena. It is therefore essential that children receive continuous assistance from their parents in the utilization of schemas, both during and after the intervention period. Consequently, parents are engaged in cognitive behavioral therapy through their participation in parent-and-child sessions. The recommendations outlined below for sessions with children can also be applied to parent-and-child sessions (Matthys & Schutter, 2024). It is proposed that the three schemas are addressed with the child during the cognitive behavioral sessions on the social problem-solving steps: Interpretation, clarification of goals, generation of solutions, evaluation of solutions, and response decision.

Discussing goals in sessions with children can help them in coming up with appropriate goals and generating adequate solutions to social problems. When introducing the topic ‘goals,’ the therapist uses the term ‘we’ (“When we have a problem …”) in order to avoid a defensive reaction from the child; this is critical especially for adolescents who may feel more defensive than children. “When we have a problem, it may be helpful to ask what is our goal: What is the goal we are aiming at? What do we want to accomplish? If we know our goals, we can better think about what to do.” This Socratic questioning approach can be used to broaden children’s thinking and to access new knowledge. Socratic questions ignite children’s curiosity and wonder. Instead of offering interpretations to children, Socratic questions aim to help children arrive at their own interpretations (McLachlan et al., 2016). In view of making Socratic questions accessible for children, it is recommended to ask short, simple open-ended questions and avoiding why questions (Friedberg & McClure, 2015; McLachlan et al., 2016). Socratic questioning is used in order to find out characteristic goals of the child (e.g., What would you want to achieve in that situation?), encourage reflection on these goals (What would that lead to?), and explore alternative goals that had previously been outside the child’s attention (Maybe there are other things you could accomplish?). Coming up with alternative goals can ultimately result in the generation of appropriate responses.

Discussing normative beliefs in sessions with children can help them in interpreting problem situations adequately, generate appropriate solutions, evaluate solutions, and select appropriate solutions. The therapist introduces these topics as follows: “Some children think it is okay to scream at a child when this child has said something bad first. These children think it is okay to scream at the child or to threaten to hit the child because they are convinced that’s the way people treat each other. When children think that only harsh or tough solutions work, they will decide to use these kind of solutions.” Socratic questioning can be used in order to find out characteristic normative beliefs of the child and encourage reflection on these beliefs (Friedberg & McClure, 2015; McLachlan et al., 2016). For example, when another child has done something bad to this child, and the child is convinced that the other child did that on purpose because that is the way people treat each other, the therapist can ask: “Are you sure about that?” “How can you be so sure?” “Is another explanation possible?” “Could it have happened by accident?”.

Considering outcome expectations in sessions with children can help them in generating and evaluating solutions, and select an appropriate solution. The therapist introduces these topics as follows: “After you have come up with solutions to the problem you can ask questions such as: ‘What do I think will happen if I do or say that? Will that help solve the problem? What is the direct effect for myself and for the other? And what is the effect in a week or a month? Do I not harm the other person with this solution? Is it correct to do that?’ It is indeed important to think about the consequences of your behavior, in the short and long term.” Socratic questioning can be used in order to find out characteristic outcome expectations of the child and encourage reflection on these outcome expectations (Friedberg & McClure, 2015; McLachlan et al., 2016). For example, the therapist can open a discussion as follows: “There are tough solutions to a social problem. What are the consequences of tough solutions?” The therapist then asks the question: “There are also kind solutions to social problems. Let us talk about kind solutions and see if they are likely to work or not.”

The three schemas discussed in the sessions all have a moral character. Morality is about values such as the well-being of others, the rights of others, honesty, and justice. These moral values become visible in normative behavior such as helping others. Moral values (e.g., the well-being of others) underlie goals (e.g., relationship building versus revengeful goals), as well as outcome expectations (e.g., concern about the other’s distress versus not worrying about harming others) and normative beliefs (e.g., kind solutions versus aggression solutions work). In discussions with the child about moral issues there is a risk that a punitive approach by adults prevails. When adults talk to their children in punitive ways this diminishes children’s other-orientation by leading them to focus on their own negative affect (Recchia & Wainryb, 2023). As a result, children are less likely to explore the psychological facets of their experiences that account for their actions (Recchia & Wainryb, 2023), such as their goals, outcome expectations, and normative beliefs. Therefore, therapists create a climate in which children are encouraged to talk about their experiences and related thoughts and feelings, both in group sessions with children and in parents-and-child sessions. Finally, given the importance of positive social experiences precipitated in the three schemas, it may be necessary during the course of cognitive behavioral therapy to add behavioral parent training booster sessions when therapists notice a relapse of negative social experiences at home, at school, or in the neighborhood. 

## Conclusion

Schemas help understand the impact of aggressive children’s social experiences on their social information processing and, ultimately, their behavior. Extensive research has shown differences between aggressive and typically developing children in goals, outcome expectations, and normative beliefs. More longitudinal studies are needed on the relationships between adverse childhood experiences, the development of atypical schemas, social information processing, and aggression in children. Although there is evidence that normative beliefs influence social information processing and behavior, no research has been conducted on the role of goals and outcome expectations in this context. It seems both appropriate and feasible to integrate schemas into cognitive behavioral therapy with the child. As shown in Fig. 1, we recommend that goals, outcome expectations, and normative beliefs be addressed with the child during the cognitive behavioral sessions on the social problem-solving steps: Interpretation, clarification of goals, generation of solutions, evaluation of solutions, and response decision. In view of the child’s use of the social problem-solving skills and associated schemas in their daily lives, it is imperative to engage the parents in the intervention process. The recommendations outlined for sessions with children may also be extended to parent-and-child sessions. 

## Data Availability

No datasets were generated or analyzed during the current study.
